# Cholesterol 25‐hydroxylase genetic deletion increases amyloid plaque load and peri‐plaque microglia and astrocytes in female APP/PS1 mice

**DOI:** 10.1002/alz.095334

**Published:** 2025-01-09

**Authors:** Justin M Long, Ramya Chengalvala, Sheryl Eveland, Danira Toral‐Rios, Anil G Cashikar, Steven M. Paul, David M. Holtzman

**Affiliations:** ^1^ Washington University School of Medicine in St Louis, St Louis, MO USA; ^2^ Hope Center for Neurological Disorders, St Louis, MO USA; ^3^ Knight Alzheimer Disease Research Center, St Louis, MO USA; ^4^ Hope Center for Neurological Disorders, Washington University School of Medicine, St. Louis, MO USA; ^5^ Washington University School of Medicine in St Louis, St. Louis, MO USA; ^6^ Knight Alzheimer Disease Research Center, St. Louis, MO USA

## Abstract

**Background:**

Neuroimmune activation plays a critical role in the pathogenesis of Alzheimer disease (AD). 25‐hydroxycholesterol (25‐HC) is a cholesterol‐derived immune‐active oxysterol produced almost exclusively by microglia within the CNS through the enzymatic activity of cholesterol 25‐hydroxylase (CH25H). 25‐HC is a potent modulator of the innate immune response, with excessive production reported to contribute to neuroinflammation and neurodegeneration in certain CNS disease models. CH25H transcript levels are upregulated in disease‐associated microglia observed in mouse models of Alzheimer‐like pathology and in human AD brain specimens. We have recently demonstrated that 25‐HC is a key driver of neurodegeneration in a mouse model of tau‐mediated neurodegeneration. Here we investigated whether 25‐HC modulates the pathological response to amyloid deposition in APPPS1‐21 (APP/PS1) mice.

**Method:**

APP/PS1 mice were crossed to CH25H knockout (KO) mice to generate male and female APP/PS1 and APP/PS1‐CH25H KO mice. Mice were collected at 5.5 months of age, a time point at which early plaque deposition is present. Mice were perfused and hemibrains fixed and sectioned. Tissue sections were stained using standard immunohistochemical techniques and imaged using whole slide brightfield imaging, wide‐field fluorescence imaging, and confocal microscopy.

**Result:**

We found that female APP/PS1‐CH25H KO mice had higher levels of total amyloid and fibrillar plaque deposition in the hippocampus and thalamus relative to female APP/PS1 mice expressing CH25H. Female APP/PS1‐CH25H KO mice also had higher levels of peri‐plaque Iba1‐, CD68‐, and GFAP‐positive staining voxels in hippocampus and thalamus compared to APP/PS1 mice, suggesting a higher level of peri‐plaque astrogliosis and microgliosis in the absence of CH25H expression. Similar trends were observed in cortex though not significant. No differences were noted between male APP/PS1 and APP/PS1‐CH25H KO mice.

**Conclusion:**

Our results demonstrate that CH25H and 25‐HC acts to constrain amyloid plaque deposition and glial responses in female APP/PS1 mice. Therefore, this pathway may represent a novel therapeutic target in AD, though further studies are needed to better understand the observed sex differences and underlying mechanism.

